# Making the cut on caesarean section: a logistic regression analysis on factors favouring caesarean sections without medical indication in comparison to spontaneous vaginal birth

**DOI:** 10.1186/s12884-023-06070-x

**Published:** 2023-10-27

**Authors:** Anja Y. Bischof, Alexander Geissler

**Affiliations:** University of St. Gallen, School of Medicine, Chair of Health Care Management, St. Jakob-Strasse 21, St. Gallen, 9000 Switzerland

**Keywords:** Birth setting, Natural birth, Caesarean section, Switzerland

## Abstract

**Background:**

In the absence of medical necessity, opting for caesarean sections exposes mothers and neonates to increased risks of enduring long-term health problems and mortality. This ultimately results in greater economic burden when compared to the outcomes of spontaneous vaginal births. In Switzerland around 33% of all births are by caesarean section. However, the rate of caesarean sections without medical indication is still unknown. Therefore, we devise an identification strategy to differentiate caesarean sections without medical indication using routine data. In addition, we aim to categorize the influencing factors for women who undergo spontaneous vaginal births as opposed to those with caesarean sections without medical indication.

**Method:**

We use Swiss Federal Statistics data including 98.3% of all women giving birth from 2014 to 2018. To determine non-medically indicated caesarean sections in our dataset, we base our identification strategy on diagnosis-related groups, diagnosis codes, and procedure classifications. Subsequently, we compare characteristics of women who give birth by non-medically CS and external factors such as the density of practicing midwives to women with spontaneous vaginal birth. Logistic regression analysis measures the effect of factors, such as age, insurance class, income, or density of practicing midwives on non-medically indicated caesarean sections.

**Results:**

Around 8% of all Swiss caesarean sections have no medical indication. The regression analysis shows that higher age, supplemental insurance, higher income, and living in urban areas are associated with non-medically indicated caesarean sections, whereas a higher density of midwives decreases the likelihood of caesarean sections without medical indication.

**Conclusions:**

By identifying non-medically indicated caesarean sections using routine data, it becomes feasible to gain insights into the characteristics of impacted mothers as well as the external factors involved. Illustrating these results, our recommendation is to revise the incentive policies directed towards healthcare professionals. Among others, future research may investigate the potential of midwife-assisted pregnancy programs on strengthening spontaneous vaginal births in absence of medical complications.

**Supplementary Information:**

The online version contains supplementary material available at 10.1186/s12884-023-06070-x.

## Background

The mode of birth relies on the condition of the mother and the neonate. The child is either born spontaneously, by instrumental birth, or via a caesarean section (CS). Desirable results linked to CS, such as reduced maternal or neonatal mortality or morbidity, are attainable only up to a specific national ratio [1]. No improved outcomes are evident for the remaining CS when compared to spontaneous vaginal birth (SVB). The WHO recommends a national CS rate between 10–15% [2]. CS rates vary considerably among OECD (Organization for Economic Co-cooperation and Development) countries, which are mainly high-income and developed countries from various regions of the world [3]. However, if unified guidelines for the indication of CS are applied or a second opinion is mandatory, the variance across and within countries is expected to be lower [4, 5]. One suggested approach is the Robson classification, where every pregnant woman is assigned into one of ten clusters based on pregnancy-related characteristics, aiming to uncover reasons and outcomes for different birth modes [6, 7].

In this paper, we categorize CS into two primary groups: planned and emergency. Planned CS represents a planned intervention, which may be conducted upon or without medical indication. Women might prefer a planned, non-medically indicated CS (referred to as non-medically indicated CS from now on) due to social norms, emotional or personal experiences [8, 9]. During the 1990s, medical indication was among the primary factors guiding the decision to opt for CS [10]. However, in the present day, psychosocial elements like birth-related fear or maternal preference without concurrent medical reasons have taken precedence at the top of the list [9, 11]. Thus, the rationale for planned CS as the eligible mode of birth is no longer only due to medical indication [8, 9, 12, 13]. In contrast, emergency CS are performed as an immediate or urgent intervention once labour has commenced.

Media reports presenting an inappropriate image of CS reduce women’s perception of the associated risks [14]. Nevertheless, different studies draw attention to the risks of non-medically indicated CS [12, 15–17]. The mother faces higher risks such as higher blood loss [18], increased probability of a stillbirth [19], or negative implications for future pregnancies [16, 19], whereas the neonate is confronted by increased respiratory morbidity [19], and higher risks of short- and long-term morbidity in general [19–21] compared to SVB. Conducting a non-medically indicated CS not only increases the potential iatrogenic harm for mothers and neonates [11, 19], it also leads to increased financial burdens on the healthcare system [18, 22].

In this paper, we present a novel approach on identifying cases of non-medically indicated CS in routine data by using Swiss hospital claims data from 2014 to 2018. We examine factors that are associated with a higher likelihood of giving birth via CS in absence of any medical indication.

## Data and methods

### Data and selection of cases

The study is based on the national dataset “Medical Statistics of Hospitals” which includes all women having given birth in Swiss hospitals between 2014 to 2018 (98.3% of all births, *n* = 430,920). The dataset is provided by the Federal Statical Office containing claims data of Swiss hospitals. As neither a corresponding diagnosis-related group (DRG), procedure classification (represented by the Swiss operation classification (CHOP)), nor a diagnosis code (ICD-10-GM) is available for non-medically indicated CS, we estimate the number of non-medically indicated CS based on various criteria. Nevertheless, the dataset does not provide information about whether a woman expresses a preference for giving birth via CS or not.

**Fig. 1:**
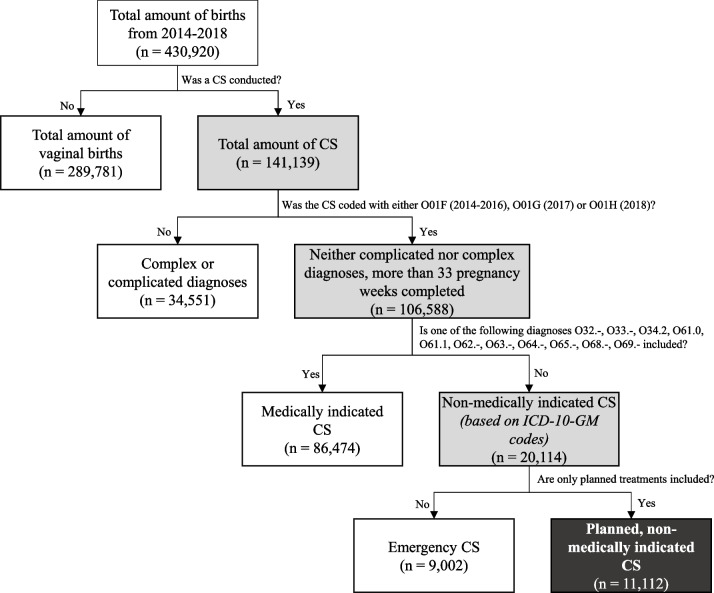
Selection process of non-medically indicated CS based on a defined set of evaluation criteria Legend: *CS* Caesarean section, *ICD-10-GM *International Statistical Classification of Diseases and Related Health Problems, 10. Revision, German Modification Annotation: The boxes represent the individual stages of how we identify non-medically indicated CS. The figure starts at the top while getting more granular from going top left to bottom right. At each box, two arrows are allocated which either affirm or deny the corresponding question. The second question about whether a CS was classified as O01F (2014–2016), O01G (2017), or O01G (2018) uses the classification of Swiss DRG (diagnose-related groups) of the corresponding year. The third question considers the diagnoses of the ICD-10-GM. The last question is about planned and emergency CS. The CHOP (Swiss operation classification) catalogue distinguishes between these two types of CS. Coloured boxes represent the path on how to get to the total number of CS without medical indication

We obtain cases of CS without medical indication by data selection as represented in Fig. 1. First, only cases with diagnosis-related groups (SwissDRG) O01F (2014–2016), O01G (2017), and O01H (2018) are considered as they refer to *“sectio caesarea, pregnancy duration more than 33 completed weeks (without complicating diagnosis, without a complex diagnosis)”*. We assume that having completed 33 weeks of pregnancy allows for spontaneous birth without fearing negative effects neither for the mother nor the neonate – every birth before 33 completed weeks classifies as extremely (less than 28 weeks) or very (28 to 32 weeks) preterm birth [23]. Furthermore, the dataset contains information about reimbursement rates, but it lacks details about actual incurred costs.

Second, diagnoses that do not allow for vaginal birth such as malpresentation of the foetus, maternal hip anomalies, or women with indicated labour who later change to an emergency CS are excluded from the dataset (see Additional file 1). We base our filtering process on Henry et al. [24] who applied the same procedure to ICD-9 codes. Subsequent studies further refine and augment this approach [25–27]. We translate the identified procedures to ICD-10-GM, which is the standard in Switzerland.

Third, we refer to the procedure classification to eliminate all CS characterized by emergency treatment features (specified by the CHOP catalogue, see Additional file 2). Moreover, the analysis begins from the year 2014 because the procedure classification did not differentiate between planned and emergency CS in prior years. This lack of distinction resulted in an inaccurate count of non-medically indicated CS.

We identify SVBs without complicated or complex diagnoses or accompanying factors via the DRG code O60D (2014–2018). The less complex DRG codes for CS are O01F (2014–2016), O01G (2017), and O01H (2018). We hypothesize that women with a CS in absence of any medical indication could have given birth spontaneously instead. This makes the group of SVB comparable to non-medically indicated CS.

Furthermore, we integrate supplementary data sources to enrich the explanatory power of the present study. Information is collected for per capita net income and nursing staff per hospital bed, both at the cantonal level and on an annual basis. For the identification of differences in the level of urbanity, we use information from the statistical atlas of Switzerland. Additionally, the Swiss society of midwives provides information on the number of practicing midwives per canton.

### Empirical strategy

We provide descriptive statistics to illustrate the characteristics of the two birth mode groups. Secondly, to analyse significant differences between the mean values of mothers giving birth by non-medically indicated CS versus SVB, we use independent-samples t-test. Thirdly, logistic regression analysis with a 5% significance level identifies the impact of factors associated with a higher likelihood of giving birth by CS without medical indication. The regression includes the following independent variables: age, insurance status, urbanity level (i.e., rural, suburban, and urban), number of midwives per 1,000 births, number of nursing staff per hospital, and income per capita.

Logistic regression analysis especially suits this research as only two outcomes are assumed: non-medically indicated CS and SVB. Furthermore, the logistic regression calculates the impact of the chosen independent variables on the chance of giving birth by non-medically indicated CS. A higher log odds ratio indicates a higher chance of giving birth by non-medically indicated CS. Similar approaches were already taken by previous studies for assessing the impact of various non-medical factors on non-medically indicated CS [12, 28]. We conduct all analyses with the Statistical Package for Social Sciences (SPSS), Version 27 [29].

## Results

### Descriptive statistics

The average number of annual births in Swiss hospitals from 2014 to 2018 was approximately 86,200 (see Table 1). Among them, 33% were born by CS and on average approximately 2,200 CS without medical indication have been detected. These amounts to almost 8% of all CS and to more than 2% compared to the total number of births in Swiss hospitals. These numbers are stable over time. In contrast, SVB without complicated or complex diagnoses faced some fluctuations. The highest rates were reached in 2014 and 2015 with almost 40,000 births. Compared to this, around 10,000 births less were given birth vaginally in 2018. The total number of detected non-medically indicated CS added up to 11,112 and 176,908 women had a SVB between 2014 and 2018. On average, 41.1% of all births were given by SVB according to the DRG code O60D.

**Table 1:** Number of total births, caesarean sections and spontaneous vaginal births in Switzerland per year

**Year**	**Total births** (# births)	**Caesarean section** (# CS)	**Non-medically indicated CS** (# nmi CS)	$\frac{\#\mathbf{C}\mathbf{S}}{\#\mathbf{b}\mathbf{i}\mathbf{r}\mathbf{t}\mathbf{h}\mathbf{s}}$	$\frac{\#\mathbf{n}\mathbf{m}\mathbf{i}\mathbf{C}\mathbf{S}}{\#\mathbf{C}\mathbf{S}}$	$\frac{\#\mathbf{n}\mathbf{m}\mathbf{i}\mathbf{C}\mathbf{S}}{\#\mathbf{b}\mathbf{i}\mathbf{r}\mathbf{t}\mathbf{h}\mathbf{s}}$	**SVB** (# SVB)	$\frac{\#\mathbf{S}\mathbf{V}\mathbf{B}}{\#\mathbf{b}\mathbf{i}\mathbf{r}\mathbf{t}\mathbf{h}\mathbf{s}}$
2014	84,438	28,324	2,206	33.54%	7.79%	2.61%	39,654	46.96%
2015	85,847	28,473	2,340	33.17%	8.22%	2.73%	39,941	46.53%
2016	87,255	28,768	2,331	32.97%	8.10%	2.67%	33,662	38.58%
2017	86,501	27,801	2,145	32.14%	7.72%	2.48%	32,701	37.80%
2018	86,879	27,773	2,090	31.97%	7.53%	2.41%	30,950	35.62%
Average	**86,184**	**28,227**	**2,222**	**32.76%**	**7.87%**	**2.58%**	**35,381**	**41.10%**
Total	**430,920**	**141,139**	**11,112**	**-**	**-**	**-**	**176,908**	**-**

Legend: *SVB* Spontaneous vaginal birth

Annotation: Total births indicate all births in Switzerland per year – independent of birth mode. The number of CS represents all CS in Switzerland per year. In non-medically indicated CS, only the defined DRG-groups O01F (2014–2016), O01G (2017) and O01H (2018) are included. Spontaneous vaginal birth displays the number of births classified by the DRG-group O60D (2014–2018)

In terms of demographics (see Table 2), the age category 30–34 years is the largest group for both types of birth modes. This is followed by the category 35–39 years for non-medically indicated CS and 25–29 years for SVB. Thus, the average age tends to be higher at non-medically indicated CS compared to SVB. Furthermore, also differences in the living area of women were observable. The distribution of women’s living areas was somewhat focused on some regions for non-medically indicated CS (Zurich followed by Espace Mittelland and North-Western Switzerland), while the rates of SVB were more equally distributed across the country.

**Table 2:** Comparison of ordinal factors between non-medically indicated caesarean section and spontaneous vaginal birth

	**Non-medically indicated caesarean section**		**Spontaneous vaginal birth**	
	**Number**	**%**	**Number**	**%**
**Total population**	11,112	100	176,908	100
**Age category**				
15–19	89	0.8	1,355	0.8
20–24	654	5.9	14,849	8.4
25–29	2,236	20.1	47,374	26.8
30–34	3,754	33.8	69,238	39.1
35–39	3,176	28.6	37,453	21.2
40–44	1,060	9.6	6,422	3.6
45–49	128	1.2	199	0.1
**Living region**				
Eastern Switzerland	1,115	10.0	22,296	12.6
Central Switzerland	1,009	9.1	16,519	9.3
North-Western Switzerland	1,805	16.2	21,544	12.2
Ticino	324	2.9	6,626	3.7
Zurich	2,871	25.8	29,409	16.6
Western Switzerland	1,616	14.5	38,837	22.0
Espace Mittelland	2,058	18.5	38,014	21.5
Other Countries	314	2.8	3,663	2.1
**Insurance class**				
Mandatory	8,431	75.9	151,295	85.5
Semi-private	1,702	15.3	18,429	10.4
Private	979	8.8	7,184	4.1
**Urbanity score**				
Urban	6,896	67.9	102,431	63.6
Suburban	1,733	17.1	27,706	17.2
Rural	1,529	15.1	30,963	19.2

Additionally, the urbanity level indicates the kind of living region (i.e., rural, suburban, or urban). Most women with a non-medically indicated CS live in urban areas (67.9%) followed by suburban (17.1%) and rural areas (15.1%). Women with SVB live mostly in urban areas (63.3%) followed by rural (19.2%) and suburban areas (17.2%).

In Switzerland, individuals have the choice to opt for supplementary insurance coverage for inpatient medical services in addition to mandatory insurance which covers basic services. Comparing this with supplemental insurance types, i.e., semi-private or private, the insured pay higher fees for additional services such as staying in a single or double-person room, and timely-preferred treatment by a specialist. The percentage of privately insured women with a non-medically indicated CS was double of those with SVB (8.8% vs 4.1%). Further, women with non-medically indicated CS were less often solely mandatorily insured than those with SVB (75.9% vs 85.5%).

DRG cost weights determine the payment of each inpatient case. Non-medically indicated CS were covered on average with a cost weight of 0.864 in 2014 remaining at the same level in 2016 and then decreasing to 0.791 in 2018. SVB showed a lower cost weight, which was 0.581 in 2014 decreasing to 0.545 in 2016 and followed by a slight increase to 0.564 in 2018. Therefore, SVB payment was generally lower compared to non-medically indicated CS across all years.

Some differences in the characteristics of the mothers belonging to either group of birth modes were already observable. Table 3 summarizes the main differences in metric variables for non-medically indicated CS and SVB. Women with non-medically indicated CS live in cantons with a lower relative rate of midwives. On the contrary, the amount of nursing staff at the hospital calculated per bed is slightly higher for women with non-medically indicated CS. Finally, income per capita is on average more than CHF 1000 higher per year for women with non-medically indicated CS in contrast to women who gave birth spontaneously. Income per capita and the insurance status show a positive correlation (r_S_ = 0.155, *p* < 0.01), i.e., woman with a higher income tend to have supplemental insurance. All variables differ in their mean values with statistical significance (*p* < 0.001) between the two birth modes.

**Table 3:** Independent samples t-test for metric variables between women with non-medically indicated caesarean sections and spontaneous vaginal births

	**Non-medically indicated CS**	**Spontaneous vaginal birth**	***p*-values**
Number of midwives per 1,000 births	37.28	37.77	< 0.001
Number of nursing staff per hospital bed	3.70	3.64	< 0.001
Income per capita	CHF 38,273.26	CHF 37,152.96	< 0.001

### Regression results

For the logistic regression (see Table 4), age category, insurance status, urbanity level, number of midwives per 1,000 births, number of nursing staff per hospital bed and income per capita were included as independent variables. All variables show the highest significance level (*p* < 0.001). A lower age category decreases the risk of giving birth by CS without medical indication, whereas the category 45–49 years served as a reference category. Additionally, for women with mandatory or semi-private insurance the odds ratio of giving birth by non-medically indicated CS is significantly lower compared to women with private insurance (reference category). For women in urban areas, the likelihood of a CS without medical indication increases compared to women in rural areas (reference category). Furthermore, a higher number of practicing midwives per 1,000 births reduce the log odds ratio by 0.019. The number of nursing staff per bed is also associated to the birth mode. The more nursing staff available per bed, the higher the chance of undergoing a non-medically indicated CS. Lastly, income per capita impacts the decision on the birth mode. A CHF 10,000 increase in annual income per capita raises the log odds ratio by 0.168.

**Table 4:** Log odds ratio for factors associated with a non-medically indicated caesarean section

Variable	Odds Ratio	Confidence Interval
**Intercept**	0.800 ***	(0.461–1.139)
**Age category (years)**		
15–19	-2.172 ***	(-2.503 – -1.841)
20–24	-2.533 ***	(-2.784 – -2.281)
25–29	-2.500 ***	(-2.742 – -2.259)
30–34	-2.413 ***	(-2.653 – -2.173)
35–39	-2.020 ***	(-2.260 – -1.779)
40–44	-1.373 ***	(-1.620 – -1.125)
**Insurance status**		
Mandatory	-0.713 ***	(-0.791 – -0.636)
Semi-private	-0.360 ***	(-0.450 – -0.270)
**Urbanity level**		
Urban	0.152 ***	(0.093 – 0.210)
Suburban	0.176 ***	(0.105 – 0.248)
**Number of midwives per 1,000 births**	-0.019 ***	(-0.023 – -0.016)
**Number of nursing staff per hospital**	0.243 ***	(0.195 – 0.291)
**Income per capita (per CHF 10,000)**	0.168 ***	(0.133 – 0.203)
**AUC**	0.625	

Significance levels: *** *p* < 0.001, *AUC* Area under the curve

*Legend*: A higher log odds ratio indicates a higher chance of giving birth by non-medically indicated CS. The following categories served as reference categories for categorical variables: 45–49 years (age), private (insurance status), rural (urbanity level)

The predictive power of the logistic regression is calculated by a ROC curve as it displays the trade-off between the detection of true positives and avoiding false positives based on the women’s characteristics. ROC curves are also suitable for imbalanced data sets, as the group sizes between non-medically indicated CS and SVB (11,112 vs 176,908) is highly imbalanced. The performed logistic regression has an area under the curve (AUC) of 0.625, which indicates an acceptable quality of the model regarding the aim of this research.

## Discussion

This paper aims to identify the percentage of non-medically indicated CS in Switzerland and second, to uncover levers for a change in the birth practice. Both questions are analysed based on a large Swiss dataset. The share of non-medically indicated CS amounts to almost 8% of all CS, which are more than 2,200 births annually in absolute terms. Research from the neighbouring country Germany reports one out of ten CS as not medically indicated. Additionally, the average non-medically indicated CS ratio in Germany is around 1% of the total number of births [30]. Other comparable studies only controlled for one diagnosis for identifying non-medically indicated CS [12, 24]. However, the presented approach allows for a more precise approximation of the number of CS without medical indication due to the holistic range of evaluation criteria.

The DRG cost weight for non-medically indicated CS generates higher payments compared to SVB. Reasons for this imbalance are the costs of expensive infrastructure (such as operating rooms and hospital bed charges) and higher personnel requirements (such as interdisciplinary operation teams). On the other hand, a higher level of uncertainty exists for SVB. On the contrary, there is a greater degree of uncertainty associated with SVB as it is inherently less predictable and demands higher time and personnel resources. Furthermore, resources must be kept available such as additional staff for an emergency CS or operating room capacities although they might not be used. Hence, for future infrastructure (hospital) planning, also the non-usage of available resources needs to be considered when setting payment rates.

The average age of the childbearing mother does not significantly differ between the two investigated modes of birth. The largest age category is at both birth modes 30–34 years. However, for non-medically indicated CS the group of 35–39 years follows whereas for SVB the category of 25–29 years ranks second. Hence, women with non-medically indicated CS are in general slightly older than women having given birth by SVB. This is also supported by previous research [28, 31] which found that women with non-medically indicated CS tends to be older than women with SVB. This can be explained as age is a factor associated with a higher risk for complications at birth [18, 32].

Further, a difference in insurance status of the included women exists. A higher ratio of women with semi-private or private insurance is observable in the non-medically indicated CS group compared to SVB. Similar findings were also reported by Womack et al. [31] for the United States. Additionally, a positive correlation between the income per capita and the insurance status is visible. The logistic regression further strengthened the assumption that higher income and supplemental insurance increases the likelihood of non-medically indicated CS over SVB. Faisal-Cury & Menezes [13] argued that income only impacts the decision on the mode of birth in low-income countries. However, this holds also true for developed countries such as Switzerland as shown in this paper.

The number of midwives per 1,000 births per canton might have an impact on the likelihood of a woman being accompanied by a midwife during pregnancy. Research showed that women who benefit from a midwife-led care program are more likely to give birth spontaneously [33], face better outcomes and have a positive maternal experience [11]. Contrastingly, another study claimed that midwife-led care models rather lead to an CS increase in high-resource countries [34]. Nevertheless, the present results support the first findings as women living in a canton with more midwives have a higher chance of SVB. Furthermore, logistic regression indicates that the probability of a non-medically indicated CS is lower in cantons with a higher number of midwives, as every one-unit increase in the number of midwives reduces the log odds ratio by -0.018. This leads to the assumption that the presence of midwives has a positive impact on the mother’s preferred mode of birth.

The paper identifies decisive factors for the decision on the mode of birth. To the authors’ knowledge, this is the first paper that chooses the presented approach to identify non-medically indicated CS in Switzerland, as corresponding procedures, diagnoses or DRG codes do not exist. Age, number of nursing staff per bed, and income per capita are significantly higher for women with non-medically indicated CS and these factors also increase the log odds ratio of a CS, although no medical indication is present. On the other hand, women also show significant differences in the insurance class, urbanity score and the number of midwives per canton. Having mandatory insurance, living in rural areas or in areas with a high number of practicing midwives per 1,000 births decreases the chance of CS without medical indication.

As every empirical study, also this analysis faces some limitations. First, there is no classification code for non-medically indicated CS, whereby only an approximation of the number of non-medically indicated CS cases is possible. Second, despite the large amount of cases of a national dataset, the analysed data does not contain personal (such as personal beliefs, fear of birth or maternal request) nor interpersonal (such as care during pregnancy or obstetrician’s tendency) factors. Additionally, clinical information is not available and it is unknown for example whether the woman is nulliparous, primiparous, or multiparous. Therefore, it is not possible to distinguish whether a non-medically indicated CS follows a previous CS. Having such variables available would improve the predictive power of the ROC curves for the logistic regression model. Furthermore, a mandatory nationwide collection of the information specifying the classification according to Robson’s proposition would further strengthen the explanatory power of research on uncovering reasons and outcomes for different birth modes in Switzerland.

## Conclusions

We develop a strategy for identifying non-medically indicated CS in routine data in absence of any unique identifying factor. CS without medical indication can be determined based on a composition of DRGs, procedure classifications and diagnoses. Thereby, this study reveals that at least 8% of CS were on a non-medically indicated basis between 2014 and 2018. Factors significantly associated to non-medically indicated CS are higher age, supplemental insurance, higher income, more available staff at the hospital and living in urban areas. In contrast, living in an area with a higher density of practicing midwives significantly decreases the likelihood of giving birth by non-medically indicated CS, and thereby increases the likelihood of giving birth spontaneously. Health policy implications derived from this research are fourfold: first, obstetricians should be sensitized to special characteristics of women undergoing CS in absence of any medical indication and be encouraged to communicate the risks for both the mother and the neonatal more explicitly; second, the density of practicing midwives should be increased in case they can reduce the number of non-medically indicated CS and further promote the benefits of spontaneous birth; third, the DRG cost weights for non-medically indicated CS and SVB should be aligned to avoid inadequate incentive mechanisms to prefer non-medically indicated CS over SVB due to higher reimbursement rates. Giving birth to a child is different from typical treatments provided in a hospital, as personal beliefs and preferences must be taken into consideration.

### Supplementary Information

**Additional file 1.** Diagnoses used to exclude cases from the not medically indicated CS dataset.

**Additional file 2.** Treatments used to exclude cases from the not medically indicated CS dataset.

## Data Availability

Data used for analyses, and the analytic code are available from the corresponding author on request.

## Abbreviations

AUC: Area under the curve
CHF: Swiss Franc
CHOP: Swiss operation classification
CS: Caesarean section
DRG: Diagnosis-related group
ICD: International classification of diseases
OECD: Organization for Economic Cooperation and Development
ROC: Receiver operating characteristic
SVB: Spontaneous vaginal birth
WHO: World Health Organization
