# Supplementary material for: Making the cut on caesarean section: a logistic regression analysis on factors favouring caesarean sections without medical indication in comparison to spontaneous vaginal birth
Source: BMC Pregnancy Childbirth. 2023 Oct 27;23:759. doi: 10.1186/s12884-023-06070-x (PMC10605562; doi:10.1186/s12884-023-06070-x)
Supplement: Supplementary file 1 — Additional file 1. Diagnoses used to exclude cases from the not medically indicated CS dataset. [file 12884_2023_6070_MOESM1_ESM.pdf]

Additional file1: Diagnoses used to exclude cases from the not medically indicated CS dataset

**ICD-10-GM codes**	**Definition**
O32.-	Care of the mother in the event of a detected or suspected positional and attitude anomaly of the fetus
O33.-	Care of the mother in case of identified or suspected mismatch between fetus and pelvis
O34.2	Care for the mother with uterine scarring due to previous surgery
O61.0	Failed medical induction of labor
O61.1	Failed instrumental induction of labor
O62.-	Abnormal labor
O63.-	Protracted birth
O64.-	Obstruction of birth due to positional, postural and attitude anomalies of the fetus
O65.-	Obstruction of birth due to anomaly of the maternal pelvis
O68.-	Complications of labor and delivery due to fetal distress [fetal danger]
O69.-	Complications of labor and delivery due to umbilical cord complications
