# Supplementary material for: Making the cut on caesarean section: a logistic regression analysis on factors favouring caesarean sections without medical indication in comparison to spontaneous vaginal birth
Source: BMC Pregnancy Childbirth. 2023 Oct 27;23:759. doi: 10.1186/s12884-023-06070-x (PMC10605562; doi:10.1186/s12884-023-06070-x)
Supplement: Supplementary file 2 — Additional file 2. Treatments used to exclude cases from the not medically indicated CS dataset. [file 12884_2023_6070_MOESM2_ESM.pdf]

Additional file 2: Treatments used to exclude cases from the not medically indicated CS dataset

**CHOP codes**	**Definition**
74.0X.20	Classic cesarean section, emergency
74.1X.20	Deep cervical cesarean section, emergency
74.2X.20	Extraperitoneal cesarean section, emergency
74.4X.20	Cesarean section of a different type, emergency
74.99.20	Other unspecified types of cesarean sections, emergency

CHOP = Swiss operation procedures
